# The PPARα Regulation of the Gut Physiology in Regard to Interaction with Microbiota, Intestinal Immunity, Metabolism, and Permeability

**DOI:** 10.3390/ijms232214156

**Published:** 2022-11-16

**Authors:** Maja Grabacka, Przemysław M. Płonka, Małgorzata Pierzchalska

**Affiliations:** 1 Department of Biotechnology and General Technology of Foods, Faculty of Food Technology, University of Agriculture, ul. Balicka 122, 30-149 Cracow, Poland; 2 Department of Biophysics, Faculty of Biochemistry, Biophysics and Biotechnology, Jagiellonian University, ul. Gronostajowa 7, 30-387 Cracow, Poland

**Keywords:** peroxisome proliferator-activated receptor α, leaky gut syndrome, Paneth cells, *Firmicutes*, *Bacteroidetes*, *Akkermansia muciniphila*, inflammation, mucosal immunity, nitric oxide

## Abstract

Peroxisome proliferator-activated receptor alpha (PPARα) is expressed throughout the mammalian gut: in epithelial cells, in the villi of enterocytes and in Paneth cells of intestinal crypts, as well as in some immune cells (e.g., *lamina propria* macrophages, dendritic cells) of the mucosa. This review examines the reciprocal interaction between PPARα activation and intestinal microbiota. We refer to the published data confirming that microbiota products can influence PPARα signaling and, on the other hand, PPARα activation is able to affect microbiota profile, viability, and diversity. PPARα impact on the broad spectrum of events connected to metabolism, signaling (e.g., NO production), immunological tolerance to dietary antigens, immunity and permeability of the gut are also discussed. We believe that the phenomena described here play a prominent role in gut homeostasis. Therefore, in conclusion we propose future directions for research, including the application of synthetic activators and natural endogenous ligands of PPARα (i.e., endocannabinoids) as therapeutics for intestinal pathologies and systemic diseases assumed to be related to gut dysbiosis.

## 1. Introduction

Peroxisome proliferator-activated receptors (PPARs) coordinate numerous signaling pathways involved in: (1) the sensing of nutrients (fatty acids and their derivatives), (2) the metabolism of lipids, amino acids and carbohydrates, (3) the modulation of immune system and inflammatory response. Out of three PPAR isotypes, PPARα is particularly deeply involved in fatty acid transport and catabolism, including mitochondrial and peroxisomal β-oxidation and branched-chain amino acid catabolism, while it also strongly inhibits inflammation through the repression of nuclear factor kappa B (NFκB), the activation protein 1 (AP-1), as well as the signal transducer and activator of transcription (STAT) signaling pathways [1].

In this review, we shall analyze the role of PPARα in the regulation of immunological processes in gut mucosa, which potentially alter the microbiota composition and host-microbiota interactions. On the other hand, we will also discuss the impact of microbiota and its metabolites on the activity of PPARα in the gut epithelia, lymphoid cells and the systemic energy metabolism. These two directions will illustrate the reciprocal interactions between PPARα and a variety of commensal microorganisms.

Intestinal inflammation is commonly accompanied by the epithelial barrier dysfunction, frequently referred to as the “leaky gut” syndrome. The already demonstrated and theoretically feasible ability of PPARα agonists, both endogenous and synthetic to counteract processes leading to the intestinal leakage, will also be presented.

## 2. The Role of PPARα in the Gastrointestinal Mucosa: Modulation of Metabolism and Immunity

Transcriptomic analysis of *Ppara* gene in the gut revealed that its expression level in the human small intestine was even larger than in the liver, regarded as one of the most important destinations of PPARα activity [2]. PPARα is also abundant in a healthy colon [3]. The study by Bunger et al. [2] demonstrated that apart from the metabolism, PPARα also coordinates multiple processes involved in the innate and adaptive immune responses carried out by the intestinal mucosa.

The *Ppara* transcription quantification in villus-crypt axis showed that the highest expression is observed in the peaks of villi of the middle part between duodenum and ileum of murine intestine [2]. The analysis of the PPARα-activated gene clusters revealed the up-regulation of gene sets involved in acyl-CoA and fatty acid metabolism, which was expected, but it also pointed to carboxylic acid, sterol, aromatic compound metabolism and peroxisome biogenesis and organization. However, surprisingly numerous PPARα-regulated gene clusters were identified as immune response-related, namely: the presentation of exogenous peptides as antigens via MHC class II proteins, antigen processing and presentation, humoral defense. The last set of PPARα-regulated gene clusters included mitosis, apoptosis, angiogenesis and blood vessel morphogenesis, which most likely coordinate mucosa renewal and regeneration. The gene set enrichment analysis (GSEA) confirmed that the most up-regulated pathways included fatty acid metabolism, but also electron transport chain components, branched-chain amino acid degradation and bile acid synthesis. Interestingly, among the most downregulated genes were those related to immune response: complement system activation (both classical and lectin-dependent pathways), granzyme A-mediated apoptotic pathways, inflammation, B cell receptor (BCR) signaling, T cell signal transduction, FAS/CD95 signaling, as well as caspase apoptotic cascade. Ingenuity pathway analysis (IPA), which helps to visualize mutual interactions between genes and links them into a network with crucial participants shown as nodes and larger hubs, demonstrated that the biggest part of network downregulated by PPARα was concentrated around MHC class II transactivator (MHC2TA)-dependent genes, which comprised MHC genes participating in antigen presentation and processing, such as: HLA-DQA, HLA-DRB1, HLA-DMA, HLA-DMB, β-2 microglobulin, but also interferon regulatory factors (IRF1, IRF3), STAT1, CD79A, CD79B from B cell signaling and CCL5, CCL6 chemokines [2]. Taken together, these results suggest that PPARα plays a crucial role in the regulation of innate and adaptive branches of gut-associated lymphoid tissue (GALT) immune response.

Importantly, Bunger et al. [2] noticed that *Ppara* expression in the small intestine increased after the treatment with synthetic PPARα agonists, fenofibrate and Wy-16434. Such a treatment also led to the elongation of villi, but not the crypt depth. Assuming that the fatty acids, which are natural PPARα ligands, are abundantly released from dietary fats in the small intestine, they most likely stimulate PPARα receptors within enterocytes that absorb them. This mechanism could affect the presentation of dietary antigens by enterocytes, which are regarded as non-canonical antigen presenting cells (APCs). This could contribute to the development of tolerance towards dietary antigens.

### 2.1. The Role of PPARa in Immunotolerance of Dietary Antigens

Enterocytes uptake and transcellularly transport antigens from intestinal lumen and can present antigens to *lamina propria* T cells, because they express low levels of MHC class II molecules on their basolateral membranes [4,5]. This process, important for the development of tolerance to dietary antigens and to host commensal microbiota, is also crucial to maintaining the balance between GALT tolerance vs. hypersensitivity or inflammation. The immune tolerance to orally administered antigens occurs through various routes, for instance through transforming growth factor β (TGFβ)-mediated suppression, clonal T cell deletion, or frequently clonal T cell anergy [6]. T cell anergy develops when APCs lack full range of costimulatory molecules on their surface, such as B7-1/2 (CD80/CD86), ICAM-1, etc. Enterocytes do not express these costimulatory molecules [7], so their engagement in the antigen presentation normally leads to anergy of naïve CD4+ T cells [6]. CD8+ and CD4+ T lymphocyte populations both take part in the suppression of immune response to oral antigens, which is mediated by the release of IL-4, IL-10 and TGFβ with simultaneous suppression of IFNγ secreting cells [8].

Dendritic cells (DCs), present in intestinal mucosa and Peyer’s patches, are professional APCs which, in cooperation with microfold cells (M cells), determine the GALT response to antigens: either tolerance or priming [9]. In healthy mucosa, DCs express low levels of B7-1 and CD40 costimulatory molecules and play a crucial role in the development of tolerance to soluble proteins and noninvasive microorganisms in GALT [6]. PPARα receptors are expressed at high levels in immature DCs [10]. PPARα activation by fenofibrate or Wy-16434 inhibits DCs maturation (regarded as the elevated expression of costimulatory molecule genes) and suppresses DCs effector functions, such as IL-12 production, in response to pro-inflammatory signals (e.g., lipopolysaccharide, LPS or oxidized low density lipoproteins, oxLDL), thus directing DCs into a less stimulatory phenotype [10,11]. Interestingly, fenofibrate inhibited monocyte differentiation into DCs and their T cell stimulatory functions [12]. This effect was manifested by morphological changes, such as reduction in the length and number of dendrites, as well as the suppressed expression of DCs maturation markers (genes encoding CD1a, CD40 and HLA-DR) and reduction in LPS or oxLDL-triggered IL-10 and IL-12 secretion [12]. In summary, PPARα activation may contribute to maintenance of tolerogenic behavior of DCs towards antigens from the diet or commensal microbiota.

### 2.2. The Role of PPARα in Pathophysiology of Colitis

Due to its anti-inflammatory and metabolic functions, PPARα is regarded as a valuable therapeutic target in chronic colitis in its various forms, such as inflammatory bowel disease (IBD): ulcerative colitis or Crohn disease (CD). Multiple studies demonstrated the involvement of PPARα in the alleviation of symptoms and histological hallmarks in animal models of these diseases [13,14]. Recently, using an innovative artificial intelligence-based approach, Katkar and co-authors have designed and synthesized a dual PPARα/PPARγ agonist PAR5359 and proved it to be an effective candidate to treat IBD [15]. In their work, Boolean Network explorer (BoNE) computational tool was used to analyze multiple transcriptomic datasets (derived from healthy human colon, ulcerative colitis and CD samples, as well as normal murine gut and mouse colitis samples) to reveal a severe downregulation of PPARα and γ genes, as well as their signaling pathways in IBD. This discovery served to predict that simultaneous activation of PPARα and γ would decrease inflammation, tissue damage and fibrosis and help to restore the epithelial barrier [15]. Despite contradictory evidence in the literature regarding the role of PPARα and its agonists in IBD models (i.e., protective role confirmed in spontaneous model of colitis in IL-10 −/− mice [16]; in dextran sulfate sodium (DSS)-induced colitis [13,14,17,18]; in dinitrobenzene sulfonic acid (DNBS)-induced colitis [19]; but the disease exacerbation was documented by [20,21,22]), the computational approach suggested that the activation of both PPARα and γ receptors in the immune target cells, such as macrophages and DCs, would be beneficial through calming down the inflammation with simultaneous support of an adequate level of immune response [15]. The protein-protein interactome (PPI) analysis in silico indicated that PGC-1^α^ would be a common interactor between PPARα and γ after using a double ligand. Notably, both PPARα and PGC-1^α^ are necessary for the therapeutic effect of infliximab (a chimeric monoclonal IgG1 antibody against TNF), a golden standard drug effective in patients with moderate or severe IBD [14,23]. In the animal IBD model, the double PPARα/γ agonist PAR5359 significantly alleviated both *Citrobacter rodentii*- and DSS-induced colitis. This compound effectively reduced fecal bacteria load, leukocyte infiltration in the colon and the associated tissue damage. These results indicated a rapid clearance of pathogens and well-resolved inflammation, neither of which were seen in mice treated with a single PPARα or γ agonist [15]. PAR5359 was also effective in the alleviation of clinical and histological parameters of severity in DSS-induced colitis. Importantly, the simultaneous activation of both PPARα and PPARγ helped to preserve the expression of epithelial tight and adherens junction genes and achieve a proper balance of macrophage inflammatory and pro-resolving functions (M1/M2 gene signatures) [15]. The authors concluded that single PPARγ activation suppressed destructive inflammation but did not overcome the infection, whereas single PPARα activation supported pathogen elimination, but was not sufficient in resolving the inflammation.

### 2.3. The Role of PPARα in the Production of Antimicrobial Peptides and Paneth Cell Functions

The mucosal immune functions are supported by anti-microbial peptides, such as defensins. These peptides not only protect epithelium from pathogenic microbial insults but are also able to modulate functions of immune effector cells, such as macrophages. PPARα agonists, fenofibrate and gemfibrozil, dose-dependent induced expression of β-defensin 1 encoding gene in LPS-stimulated macrophages, while simultaneously downregulating Toll-like receptor 4 (TLR4), pro-inflammatory NFκB and Erk signaling, as well as chemokine CXCL2 and cytokine TNFα and IL-6 [24]. Surprisingly, these effects depended on the presence of β-defensin 1: siRNA-mediated knockdown of *DEFB1* gene abolished the anti-inflammatory action of PPARα agonists [24]. This mechanism involved a kind of an autocrine regulation because IP-depletion of β-defensin from the macrophage conditioned media abrogated the effect of gemfibrozil and fenofibrate.

PPARα involvement in β-defensin 1 gene expression is likely to be mediated by peroxisome proliferator response elements (PPRE) found in *DEFB1* gene [25]. These PPRE elements were proposed to act with PPARγ, which contribute to the proper anti-bacterial response in the colon [25]. Nevertheless, anti-microbial immunity in ileum does not depend on PPARγ [25], and PPARα seems to be a natural candidate here.

Alpha and beta defensins are important components of the innate anti-microbial defense, but the study by Ann et al. [24] suggest their broader immunoregulatory role. The main source of anti-microbial peptides in the small intestine are Paneth cells, which produce α-defensin (called cryptidin in mice), lysozyme, matrix metalloproteinase 7 and phospholipase PLA2G2 [26]. Paneth cells are important not only for bactericidal innate immunity, but also create a niche for intestinal stem cells (ISCs) in the crypts and support ISCs epithelial cell renewing potential. This latter role has been recently explored by Pentinmikko et al. [27], who discovered that a WNT inhibitor Notum, produced by Paneth cells in aged intestinal epithelium, impaired ICSs renewal and regenerating potential, both in human and murine intestines. The Paneth cell senescence leads to enhanced activity of mammalian target of rapamycin complex 1 (mTORC1), which is a known antagonist of PPARα [28]. Indeed, active mTORC1 signaling in ageing Paneth cells suppressed PPARα activity, which resulted in the increased secretion of Notum. Notum acts as a Wnt deacylase which induces dissociation of Wnt ligands (important for the maintenance of stemness) from LRP5/6-Frizzled receptors [27]. PPARα antagonist GW6471 was demonstrated to increase Notum secretion in ‘young’ Paneth cells, which led to a reduced regenerative potential of the intestinal mucosa [27]. Interestingly, a decline in fatty acid oxidation, a canonical PPARα-dependent metabolic pathway, is associated with an impaired ISCs function during aging [27]. These data indicate the indispensable role of PPARα in sustaining the renewal potential of ISCs niche in gut mucosa.

Paneth cell differentiation and bactericidal activity is also regulated by PPARβ [29]. This *Ppard* gene shows a particularly strong expression in the intestinal crypts, in the places occupied by Paneth cells. PPARβ activity is responsible for Paneth cell development and function through antagonism with Indian hedgehog signaling [29]. In the small intestine of PPARβ knock-out mice, there are significantly fewer Paneth cells than in wild type (wt) mice and they express lower levels of functional markers, such as α-defensin, lysozyme and MMP-7 (the enzyme necessary for activation of α defensins) and lysates from these intestines show reduced bactericidal activity towards *Escherichia coli* K12 [29]. Nevertheless, the abundance of bacterial populations of *Enterobacteria*, *Staphylococci* and *Bacteroides* sp. in the small intestines were similar in both *Ppard* +/+ and −/− mice. The significant differences in favor of wt mice were seen in case of *Lactobacilli*, but *Bifidobacteria* were more abundant in ileum of *Ppard* −/− mice [29]. These alterations in microbiota composition do not necessarily reveal the situation in the colon, because the small intestine microbiota is much less abundant and diverse in comparison to the colon [30]. Nevertheless, these results indicate potential involvement of PPAR receptors in the modulation of Paneth cell function, which in consequence affects various gut microbiota species.

## 3. PPARα-Mediated Modulation of Gut Microbiota Composition

### 3.1. The PPARa Influence on the Microbiota Profile

PPARα activity is necessary for the maintenance of the intestinal barrier and the development of tolerance towards gut microbiota through the suppression of Th1/Th17 inflammatory response [18]. The experimental evidence demonstrated that the lack of *Ppara* expression in the gut led to dysbiosis. Dysbiosis is a state of imbalance of the natural microbiota composition, abundance or diversity, associated with a pathological outcome. The absence of PPARα and subsequent dysbiosis facilitated the development of colitis in mouse model, due to up-regulation of the Th1/Th17 response. PPARα up-regulated IL-22 production by innate immune cells, which helped to maintain a proper intestinal mucosa function and tolerance of gut microbiota [18]. IL-22 is an IL-10 family cytokine, which is indispensable for the production of antimicrobial peptides, such as regenerating islet-derived proteins RegIIIβ, RegIIIγ, calprotectin (S100A, S100B), as well as tight junction protein claudin 2 [31,32]. These proteins are crucial for the host response to fight with the intestinal pathogens. *Ppara* −/− mice had decreased levels of IL-22 and anti-microbial peptides, but also significantly larger populations of segmented filamentous bacteria (SFB), *Prevotellaceae* and TM7 bacteria (*Saccharibacteria*) in their colons, compared to wt mice, and were much more susceptible to colitis [18] (Table 1). These results demonstrate that PPARα-mediated IL-22 production by innate lymphoid cells is necessary for maintaining gut commensal microbiota homeostasis, which means protecting from pathogens, supporting “healthy” microbiota and suppressing unnecessary inflammation.

PPARα-induced changes in the production of anti-microbial peptides in the gastrointestinal mucosa may have deep consequences to the host-microbiome homeostasis and the composition of intestinal microbiota. The example of mice with various levels of α defensins (*MMP7* −/− mice that lack mature, active α defensins and homozygous or heterozygous transgenic mice expressing human α defensin *DEFA5* gene (+/−), (+/+)) revealed that the presence of α defensin dramatically changed the microbiota composition in the small intestine, i.e., significantly increased *Bacteroidetes* and *Proteobacteria* counts, while decreased the abundance of *Firmicutes* and *Actinobacteria* [33]. Importantly, the total bacteria load in feces of the mice with all of the genetic backgrounds were the same. Interestingly, the presence of human α defensin resulted in a complete loss of segmented filamentous bacteria (SFB). SFB belong to *Firmicutes* and are capable of invading mucus. Through a direct adhesion to epithelial cells, they induce IL-17 mediated inflammatory response [34,35]. The amount of SFB in the intestines showed a strong positive correlation with the number of IL-17A producing CD4+ T cells in *lamina propria*: no IL-17A was detected in mice with no SFM, whereas mice with higher abundance of SFM presented higher IL-17A production by T lymphocytes [33]. These data point to the crucial role of the anti-microbial peptides, such as α defensin, in the gut microbiota modulation and functional profile of T lymphocytes in the mucosa. This concept points to a direction where PPARα and its agonists may be exploited in various pathophysiological conditions associated with gut dysbiosis and/or inflammation.

### 3.2. The PPARa Agonist as Modulators of Gut Microbiota Diversity

Oleoylethanolamide (OEA) is an endogenously produced PPARα ligand, classified as an endocannabinoid due to its similar structure to anandamide. Apart from PPARα, OEA activates other receptors: G-protein coupled GPR119 and GPR55 receptors, which also bind various cannabinoids, although their homology to ‘classical’ cannabinoid receptors CB1 and CB2 is not very high [36,37,38]. OEA is synthesized in the small intestine from membrane lipids N-acylated phosphatidylethanolamines (NAPEs) in response to feeding and has an anorexigenic effect [39,40] or enterocytes convert dietary oleic acid into OEA through NAPE-dependent or independent reactions [41]. A recent study by Di Paola and co-authors [42] demonstrated that the i.p. administration of exogenous OEA to mice showed a tendency to increase microbial diversity and shift in colonic microbiota composition towards higher *Bacteroidetes* and lower *Firmicutes* abundance (the comparison between T0 before the treatment and on day 11 of OEA treatment). On the genus level, the treatment decreased *Bacillus* and *Lactobacillus* (*Firmicutes*) counts, whereas increased *Bacteroides*, *Prevotella* and *Parabacteroides* (*Bacteroidetes*) representation (Table 1). The decrease in *Firmicutes/Bacteroidetes* (F/B) ratio after OEA administration mimics the effect of a polysaccharide-rich, low-fat diet. The altered microbiota composition also had implications for mucosal immune system: the cells isolated from Peyer’s patches of the OΕA treated mice were less prone to the induction of pro-inflammatory response to LPS; they produced significantly less IL-6, IL-17 and IFNγ than the cells from the control mice and shifted the T cell polarization from Th1 to Th2 type [42].

The evidence from animal and human studies indicate that low F/B ratio is associated with lean body structure (smaller proportion of adipose tissue), the process of losing weight in obese individuals [43,44,45]. A high percentage of *Firmicutes* in gut microbiota correlates with more effective calorie yield from the same amount of ingested food, which may lead to a positive balance of calories while maintaining the same energy expenditure. If such a situation lasts long enough, it may result in weight gain and obesity development [44,46].

Apart from endogenous PPARα ligands, such as OEA, synthetic agonists have also been proved to be effective in modulating gut microbiota. The study performed on mice with high-fat diet (HFD)-induced diabetes revealed that mice treated with fenofibrate (0.1% of feed supplementation) gained significantly less weight and had increased concentrations of short chain fatty acids (SCFA: acetate, propionate, butyrate) in serum, retina and feces compared to mice which did not receive the drug [47]. The elevation of SCFA suggested possible changes in the gut microbiota composition, as these fatty acids are characteristic products of undigestible polysaccharide fermentation carried out by certain species of commensal bacteria [48]. SCFA in the colon protect mucosa integrity serve as an energy source of colonocytes and calm down inflammation [49]. The microbiota composition determines the amounts of SCFA produced in the gut.

The administration of fenofibrate increased the percentage of *Bacteroidetes* and decreased *Firmicutes*, thus normalizing F/B ratio, which was severely up-regulated in HFD mice [47] (Table 1). The percentage of *Proteobacteria* group was also decreased, which is important because an abundance of these microbes is associated with endotoxemia and systemic inflammation due to properties of their LPS [47,50]. Increased counts of *Proteobacteria* in mice may result from high-fructose diet and are a marker of non-alcoholic fatty liver disease (NAFLD) [51]. LPS-mediated endotoxemia is a complication frequently associated with HFD-induced obesity because obesity induces dysbiosis, which negatively affects the epithelial tightness [52]. Importantly, a synthetic PPARα agonist fenofibrate improved barrier functions of intestinal mucosa in HFD mice, which was manifested by lower permeability for fluorescently labelled high molecular weight dextran and higher expression of the genes encoding for tight junction proteins, zonula occludens 1 (ZO-1) and occludin in the colon [47]. These data suggest that such an action of fenofibrate could counteract the development of endotoxemia. In support of this notion, Wang and co-authors demonstrated that fenofibrate downregulated TLR4 and inhibited both systemic and retinal inflammatory response, which could be attributed to the recovery from HFD-induced dysbiosis [47].

The PPARα synthetic agonist Wy-16434 has been shown to reverse dysbiosis induced by a high-fructose diet in the murine model of NAFLD, as well as in obese mice on HFD [53,54]. The authors observed a significant decrease in F/B ratio, a decreased percentage of *Proteobacteria*, an increase in *Actinobacteria* abundance in the metabolically challenged group, treated with the PPARα agonist Wy-16434, in comparison to the vehicle treated group [53,54] (Table 1). The effect of Wy-16434 was strengthened by administration of linagliptin, a synthetic inhibitor of dipeptidyl peptidase-4, which blocks proteolytic degradation of glucagon-like peptide-1 (GLP-1) [53,54]. GLP-1 is a hormone released in response to feeding that controls glucose homeostasis and inhibits food intake [55]. Moreover, Wy-16434 with linagliptin increased mucin synthesis and thickening mucus layer in the intestinal mucosa, as well as induced ZO-1 and occludin, therefore improving the gut barrier functions [53,54]. In the model of HFD-induced obesity, PPARα activation and GLP-1 stabilization resulted in decreased endotoxemia, which could be explained by the lower intestinal permeability to LPS. Decreased endotoxemia calmed down Kupffer cell-mediated inflammation in the liver [54]. The additional beneficial effect exerted by Wy-16434, linagliptin of both, was the increase in *Ppara* expression in the liver, the activation of PPARα-dependent fatty acid oxidation pathway and the attenuation of high-fructose diet-induced liver steatosis [53].

**Table 1:** The experimental evidence of PPARα-mediated modulation of gut microbiota.

Model/Agent	Outcome	Reference
*Ppara* −/− mice	Increased population of segmented filamentous bacteria (SFB), TM7 bacteria	[18]
Oleoylethanolamide (OEA), an endogenous PPARα agonist, mice	A trend of *Bacillus, Lactobacillus* decrease, *Bacteroides, Prevotella, Parabacteroides* increase, decreased *Firmicutes/Bacteroidetes* ratio	[42]
Wy-16434, a synthetic PPARα agonist, mice on high-fructose diet	Reverse of dysbiosis induced by a high-fructose diet, decreased *Proteobacteria*, increased *Actinobacteria* counts	[53,54]

## 4. Modulation of PPAR Activity by the Various Taxa of Commensal Bacteria

Lactic acid bacteria (LAB) are Gram-positive components of natural gut microbiota, which utilize carbohydrates as the main or sole source of carbon. *Lactobacillaceae*, *Leuconostocaceae* and *Streptococcaceae* are the main taxonomic groups classified as LAB and they belong to *Firmicutes* phylum. They can adhere to intestinal mucus layer, contribute to maintenance of gut barrier and exert diverse health-promoting activities through sustaining diversity of commensal microbiota while restricting the prevalence of pathogenic species, so are regarded as valuable members of the microbiota community [56,57,58]. *Lactobacilli* produce nanomolar amounts of hydrogen peroxide, which is important for the host protection from invasive pathogenic bacteria [59]. Hydrogen peroxide production by *Lactobacilli* is also responsible for protection against gut mucosa injury and the acceleration of tissue healing in DSS-induced colitis in mice [60].

Apart from these beneficial actions, *Lactobacilli* can metabolize lipids and long chain fatty acids and transform them into active mediators that modulate host metabolism and inflammation [61,62]. *Lactobacilli* possess a diverse enzymatic toolbox to generate hydroxy-, keto- or conjugated-fatty acids from dietary polyunsaturated fatty acids, such as linoleic acid, which can later be found in various host tissues and organs [63]. Interestingly, partially saturated *trans* derivatives, such as 10-oxo-11-*trans*-octadecenoic or 10-hydroxy-11-*trans*-octadecenoic acid, are also bacterial products [61,63], making disputable the belief that the *trans* isomers of fatty acids are exclusively produced during chemical fat hardening in food industry. The linoleic acid derivatives identified as the products of *Lactobacillus plantarum* (*Lactiplantibacillus plantarum* in new nomenclature, according to [64]) AKU1009a strain, such as keto- and hydroxy-octadecenoic acid species activate PPARs, particularly PPARα and PPARγ [61]. Moreover, 10-oxo-12(Z)-octadecenoic acid (αKetoA), a potent PPARγ agonist (capable of 7-fold induction in in vitro transactivation assay using a PPRE reporter plasmid), was shown to induce adipocyte differentiation, lipid accumulation, which may contribute to increased adiposity [61] (Table 2). αKetoA levels were higher in feces of mice maintained in specific-pathogen free conditions, which received *L. plantarum* and feed containing linseed oil (a rich source of α linoleic acid), in comparison to mice fed with soybean oil [62]. This metabolite was absent in the feces of germ-free mice fed with linseed oil, which suggested that αKetoA is truly a microbial product. αKetoA had an anti-inflammatory effect on macrophages and promoted their M2 polarization, as well as reduced number of adipose-tissue infiltrating macrophages in HFD-induced obesity model in mice [62]. These effects were PPAR dependent. Another linoleic metabolite, 10-hydroxy-*cis*-12-octadecenoic acid (HYA), has been shown to ameliorate DSS-induced intestinal permeability through up-regulation of tight junction proteins (occluding, ZO-1, ZO-2, claudin 1, claudin 3) [65]. HYA suppressed inflammation and improved histological parameters in the colon of DSS-treated mice, although not PPARs, but the G-protein coupled receptor GPR40 was responsible for these effects [65].

Several LAB strains, some applied in food fermentation processes, were demonstrated to induce the expression of *Ppara* and its downstream genes involved in fatty acid catabolism, which is regarded as a promising strategy for the supportive treatment in various metabolic diseases, such as NAFLD or obesity. *Lactobacillus kefiri* DH5 (*Lentilactobacillus kefiri* in new nomenclature, [64]) [66], *Lactobacillus amylovorus* CP1563 [67,68] and *Lactobacillus plantarum* (*Lactiplantibacillus plantarum*) FRT10 strains [69] induced *Ppara* expression and PPARα function in liver and adipocytes, which ameliorated the liver steatosis parameters in NAFLD or obesity in HFD mice [69]. The organic extract from *Lactobacillus amylovorus* CP1563 potently activate PPARα and γ in the in vitro transactivation assays [68]. Mill-fragmented preparations of *L. amylovorus* CP1563 were shown to increase HDL plasma concentration in mice, which serves as an in vivo manifestation of PPARα activity on the systemic cholesterol metabolism [68].

Some defined members of gut commensal microbiota exert their beneficial and health promoting effects through inducing *Ppara* expression; the mixture of *Bacillus subtilis* and *Enterococcus faecium* suppresses liver inflammation, improves intestinal barrier function and alleviates liver steatosis symptoms in NAFLD murine model through activation of hepatic PPARα [70]. The presence of a commensal microbe, *Bacteroides acidifaciens,* in the gut has been correlated with maintenance of lean phenotype in HFD mice [71]. The detailed experiments showed that *B. acidifaciens* JCM10556 strain prevents excessive adiposity and weight gain in the murine model of obesity through glucose tolerance improvement and up-regulation of PPARα activity and fatty acid oxidation in adipocytes, which resulted in the increased energy expenditure [71]. This study underscores the importance of the gut microbiota for total body energy balance and at least partially explains the relation between dysbiosis and obesity, for example the relevance of F/B ratio.

In certain situations, the direct contact between live bacteria and epithelial cells is not necessary for inducing the biological effect in gut mucosa. Some bacterial species emit extracellular vesicles (EVs) which reach cell surface and can exert biological effects. One example of such an action was demonstrated for *Faecalibacterium prausnitzii,* a prominent butyrate producer in the colon [72]. The incubation of colonic epithelial Caco-2 cells with *F. prausnitzii*-derived EVs induced a several fold increase in the mRNA for tight junction genes ZO-1 and occludin, as well as all three PPAR isotypes. Surprisingly, in the similar experiment with Caco-2 cells, the incubation with live *F. prausnitzii* cells led to a significant downregulation of ZO-1, occludin and PPARα, β and γ [72] (Table 2). The authors concluded that the *F. pracusnitzii* EVs, but not live cells, are good candidates to be used as ‘postbiotics’, i.e., the bacterial-derived products that can support intestinal barrier function and possibly exert an immunomodulatory action through the induction of PPARs. Postbiotics could be a therapeutic valuable option for patients who should not receive live bacteria as probiotics, e.g., immunocompromised individuals, patients with severely disrupted intestinal barrier, or with the risk of endotoxemia [72].

Commensal microbiota residing in the colon produce millimolar amounts of short chain fatty acids (SCFA) as a result of fermentation of undigested complex polysaccharides [73]. SCFA, including acetate, propionate and butyrate (usually present in human gut in the proportions of 60%, 20%, 20%, respectively) contribute to lowering the pH in the gut lumen, which reduces the risk of overgrowth of microbial pathogens [74]. SCFA play an important role in maintaining metabolic and immunological homeostasis in the gut mucosa. The majority of anaerobic bacteria form acetate, whereas *Bacteroides* sp. and *F. prausnitzii* belong to the main propionate and butyrate producers, respectively. Propionate was shown to potently and dose-dependently induce *Ppara* expression on mRNA and protein level in mouse colonic epithelial cells, but it also acts as PPARα ligand, triggering fatty acid catabolism through β-oxidation pathway [75]. Butyrate is a metabolite of extreme importance for gut mucosa physiology, because it is the main energy source for colonocytes [76], but it also blocks inflammatory response through the inhibition of IL-6/STAT3/IL-17 pathway and decreases proinflammatory lymphocyte T17 population while promoting an increase in Tregs number [77]. Butyrate produced by *F. prausnitzii* plays a crucial role in this process and limits bacterial population in the gut of IBD patients that alleviates the symptoms of this disease [77,78,79,80].

Obesity and poor metabolic health status correlate with lower abundance of *Akkermansia muciniphila* (*Verrucomicrobia* phylum) in the gut [81,82]. *A. muciniphila* is a Gram-negative bacterium, present in 90% of people and constitutes between 1 and 5% of total colon microbiota composition [83]. This microbe resides in the mucin layer that covers intestinal mucosa, particularly in the cecum region [84]. This microbe adheres to the surface of intestinal epithelial cells and is capable of utilizing glycans produced by host as a source of carbon, particularly when the host’s diet lacks complex polysaccharides [84,85,86]. Nevertheless, *A. muciniphila* does not destroy mucus layer but on the contrary, it supports its renewal by increasing the number of goblet cells [87]. *A. muciniphila* and its EVs strengthen gut barrier functions through up-regulating the expression of tight junction genes, occludin, claudin 4 and ZO-2 [88,89]. It is believed that through the interaction with the intestinal epithelial cells, this bacterial species contributes to the development of immune tolerance and maintenance of immune and metabolic homeostasis [84]. A recent study has demonstrated that *A. muciniphila* increases the number of RORγt+ Tregs in *lamina propria* and inhibits DSS-induced colitis through the interaction with TLR4 [90]. Indeed, a decreased count of *A. muciniphila* was reported in murine models of IBD and patients with ulcerative colitis or CD [90,91,92,93]. The experiments performed on germ free (GF) mice and the animals colonized with *A. mucuniphila* revealed that the colonization had a strong impact on the gut epithelial cell gene transcription pattern with variability in the particular regions of the gastrointestinal tract [84]. Compared to GF animals, the set of genes up-regulated by *A. muciniphila* included genes from PPARα/RXRα signaling pathway governing lipid catabolism and ketone body synthesis, genes involved in tryptophan metabolism, integrin signaling and nicotinamide metabolism in ileum and cecum region. In the colon, the genes associated with immune cells proliferation and differentiation, including B cell receptor signaling, leukocyte extravasation as well as complement and coagulation cascades, were up-regulated [84]. Collectively, these data suggest that *A. muciniphila* modulate epithelial cell metabolism and promote the development of immune tolerance.

**Table 2:** The experimental evidence that microbiota modulate PPARα signaling. The bacterial species names are taken from the original references, but in certain cases, the new species names according to the updated taxonomic nomenclature of the genus *Lactobacillus* [64] are given in parentheses.

Species/Strains	Active Form or Metabolite	Molecular Target	Reference
*Lactobacillus plantarum* (*Lactiplantibacillus plantarum*) AKU1009a	α-linoleic acid metabolites, keto- and hydroxy-octadecenoic acid species	PPARα, PPARγ	[61,62]
*Lactobacillus kefiri* (*Lentilactobacillus kefiri*) DH5	Live bacteria	PPARα	[66]
*Lactobacillus amylovorus* CP1563	Mill-fragmented bacteria	PPARα	[67,68]
*Lactobacillus plantarum* (*Lactiplantibacillus plantarum*) FRT10	Live bacteria	PPARα	[69]
*Bacillus subtilis* + *Enterococcus faecium*	Live bacteria	PPARα	[70]
*Bacteroides acidifaciens* JCM10556	Live bacteria	PPARα	[71]
*Faecalibacterium prausnitzii*	Extracellular vesicles (EVs)	PPARα, β, γ, tight junction proteins	[72]
	propionate	PPARα	[75]
*Akkremansia muciniphila*	Live bacteria, autoclaved bacteria	PPARα/RXRα	[82,84]
*Akkermansia muciniphila*	Live bacteria, 1-PG, 2-PG	PPARα	[94]
*Akkermansia muciniphila*	Pasteurized bacteria, Amuc_1100	TLR2	[95]
*Lactobacillus plantarum* (*Lactiplantibacillus plantarum*) NCIMB8826	Live bacteria	PPARα	[96]

## 5. PPARα and the Gut Microbiota Composition—Implications for Total Energy Expenditure

Certain components of gut microbiota, particularly the presence of *A. muciniphila*, not only exert a strong impact on the metabolism on the cellular level but are also able to change the systemic energy expenditure, which translates to the level of adiposity and body composition. In obese individuals or in HFD-fed obese mice, the progressive dysbiosis leads to a decreased percentage of *A. muciniphila* in gut microbiota [82]. The restoration of the diminished population of this bacterial species in obese mice increased the expression of PPARα-driven genes involved in the fatty acid catabolic program, i.e., PPARα, CPT1A, PGC-1^α^ and ACOX in adipocytes, which resulted in the improvement of the fat mass to lean mass ratio [82] (Table 2). In this study, the metabolic effects were accompanied by the recovery of the intestinal mucus layer thickness and were evoked only by live, but not heat killed (autoclaved) *A. miciniphila* [82]. Nevertheless, further work by this research group revealed that *A. muciniphila* heat-inactivation in milder, less denaturing conditions (pasteurization in 70 °C for 30 min) retained or even enhanced the positive metabolic outcome in obese mice: less body weight and fat mass gain during HFD feeding; lower glucose intolerance and lower insulin resistance, compared to HFD without pasteurized *A. muciniphila* supplementation [95]. The molecular analysis demonstrated that the presence of a relatively thermostable outer membrane protein, Amuc-1100, is responsible for exerting the beneficial metabolic effects through the activation of TLR2 signaling. Importantly, the *A. muciniphila* LPS is structurally different than the one from *Escherichia coli* and does not effectively bind to TLR4 [86]; therefore, it is less likely to induce endotoxemia. Moreover, Amuc-1100 contributes to the strengthening of the intestinal epithelial barrier through downregulation of cannabinoid receptor CB1, as previously implicated by the increased gut permeability, and by up-regulating the tight junction genes encoding claudin 3 and occludin in jejunum and ileum [95].

Molecular components of *A. muciniphila*, such as Amuc-1100, significantly decrease the energy efficiency of ingested food. The fecal energy content in the animals treated with pasteurized *A. muciniphila* was higher than in the control group, whereas the food intake remained similar in both groups [94,95,97]. Interestingly, the administration of pasteurized *A. muciniphila* also increased physical activity and decreased the value of respiratory exchange ratio (RER), a parameter that informed about the proportion of aerobic or anaerobic metabolism and the energy substrates that were preferentially utilized. Average RER oscillates around 0.8; the values near 0.7 indicate fat burning, whereas the values of 1.0–1.2 suggest that the anaerobic carbohydrate catabolism prevails. The HFD-fed mice treated with pasteurized *A. muciniphila* had lower RER than mice on the control diet, which indicated that their increased energy demand was covered mostly through mobilization of fat from adipocytes [97]. Interestingly, the administration of live and pasteurized *A. muciniphila* to obese humans significantly increased the plasma concentration of the endocannbinoid 1-palmitoyl-glycerol (1-PG), whereas 2-palmitoyl-glycerol levels were significantly increased by live bacteria, compared to the placebo group [94]. Other components of the endocannabidiome were not altered. Palmitoyl monoacylglycerols 1- and 2-PG were identified as endogenous agonists of PPARα, but not PPARγ; therefore, the beneficial effects of *A. mucuniphila* in the metabolic syndrome can be attributed to the activation of PPARα [94] (Table 2). Furthermore, a randomized clinical trial showed that the endocannabinoid OEA exogenously applied to obese individuals significantly increased the *A. muciniphila* abundance and reduced average caloric intake in comparison to the placebo treated group [98]. Taken together, these results illustrate a mutual interplay between *A. muciniphila* and PPARα agonism and encourage the application of these bacteria or their components as potential treatment against obesity.

## 6. The PPARα-Mediated Alterations in Metabolism and Their Implications to Host-Microbiota Interactions

As mentioned above, the composition of gut microbiota determines energy yield from food and, subsequently, the modes of its storage in the host. Germ-free mice are leaner than their counterparts bred in conventional conditions with the developed gut microbiota, although the energy intake is higher [46]. Commensal microbes also influence the host energy homeostasis in the situations of food deprivation. Crawford and co-authors showed that GF mice had lower plasma levels of ketone bodies than conventionally bred mice during fasting and lower liver expression of *Ppara* [99]. The lower hepatic levels of PPARα resulted in the impaired ketogenesis due to the less efficient induction of fibroblast growth factor 21 (*Fgf21*) and mitochondrial 3-hydroxy-3-methylglutaryl synthase 2 (*Hmgcs2*), the genes engaged in the ketone body synthesis [99]. Fasting induced a shift in the composition of intestinal microbiota in favor of *Bacteroidetes*, at the expense of *Firmicutes*. During food deprivation and in the absence of dietary polysaccharides, *Bacteroidetes* metabolized the host glycans present in mucus and secreted large amounts of acetate that cumulated in the gut of conventionally bred mice. This process did not occur in GF mice. In mice with microbiota, acetate was absorbed from the gut and served as the source of acetyl-CoA for the ketone body synthesis. Another pool of acetyl-CoA for ketogenesis comes from the mobilization of fat stores and hepatic β-oxidation of fatty acids. GF mice only have the latter source of acetyl-CoA, hence their ketogenesis is less efficient than in mice with intestinal microbiota [99]. The myocardium of conventionally bred mice efficiently utilized the ketone bodies during fasting for energy purposes, while the heart muscles in GF mice were forced to launch a compensatory shift towards glucose oxidation in order to sustain the proper energy level. The heart weights of the latter mice were significantly reduced [99]. These results demonstrate an important, underappreciated evolutionary advantage for the host related to the presence of commensal microbiota; the conventionally bred mice could spare their glycogen stores for other purposes, such as brain or erythrocyte energy support, and rely largely on ketone body oxidation to sustain cardiac muscle function.

The regulation of metabolism by probiotic bacteria, which involves the activation of the PPARα pathway, was also demonstrated in the gut of SIV-infected macaques, who suffered from the leaky gut syndrome induced by dysbiosis resulted from SIV infection [96]. The increased intestinal permeability, associated with reduced levels of ZO-1, occludin and E-cadherin, developed in SIV infected monkeys as a consequence of IL-1β-mediated inflammation, despite the anti-retroviral treatment (ART). The analysis of metabolome present in the gut liminal contents revealed the accumulation of short- and medium-chain fatty acids, which suggested that their catabolism through mitochondrial β-oxidation, as well as oxidative phosphorylation might be impaired [96]. However, the administration of *Lactobacillus plantarum* (*Lactiplantibacillus plantarum*) NCIMB8826-MM24 rapidly (within 5 h) restored the intestinal barrier function and potently activated *Ppara* expression in the gut epithelium and this increase was even stronger than after the treatment with fenofibrate [96]. The induction of PPARα signaling and transactivation of its downstream target genes involved in the fatty acid metabolism and electron transfer chain lead to improved mitochondrial respiration and was responsible for rebuilding of the epithelial barrier tightness [96]. These results point out to the crucial role of PPARα in the protection of the gut mucosa from the SIV-induced damage, as well as the fact that PPARα is an important molecular target of probiotics, such as *L. plantarum*.

## 7. Interplay between PPARα, Nitric Oxide Production and Gut Microbiota

Nitric oxide (NO) is one of the ten simplest stable compounds of crucial importance for bacterial microflora and mycobiota. It plays a complex and often ambivalent role in the functioning of intestines, particularly for the communication between the gut and in the rest of the organism.

Nitric oxide, its production and utilization by microorganisms, has been the important missing link between the inorganic chemistry of nitrogen and biological utilization of active forms of nitrogen. Pathogenic microorganisms, but also some gut commensals, including *Escherichia coli, Lactobacilli, Bifidobacteria* [100]), are capable of executing the denitrification and reduction in nitrite to NO and NO to nitrous oxide. While this was considered to be a rapid reaction, the stationary level of NO was still believed to be maintained at a low level, mainly depending on the level of nitrate and nitrite in the food. NO can be generated in a non-enzymatic way from nitrite in acidic pH [101]. During bacterial denitrification performed in the gut, NO appears as an intermediate. The primary substrate for this process is nitrate undergoing serial reduction through nitrite, nitric oxide, nitrous oxide, down to the gaseous nitrogen [102]. Denitrification is a variation of respiration, where the electrons are transferred not to oxygen but nitrate with a parallel production of ATP [103]. In the anoxic conditions, for instance in the colon, the most important enzymes maintaining the low, but non-zero level of NO in the denitrifying bacteria include: (i) nitrite reductase, containing heme or copper and localized in the periplasma, and (ii) heme-containing reductase of NO, generating N_2_O, localized in the cellular membrane of denitrifying Gram-negative bacteria [104]. One of the reasons for maintaining low levels of NO by bacteria is its extreme toxicity, another reason for the denitrification is ATP production.

**Figure 1:**
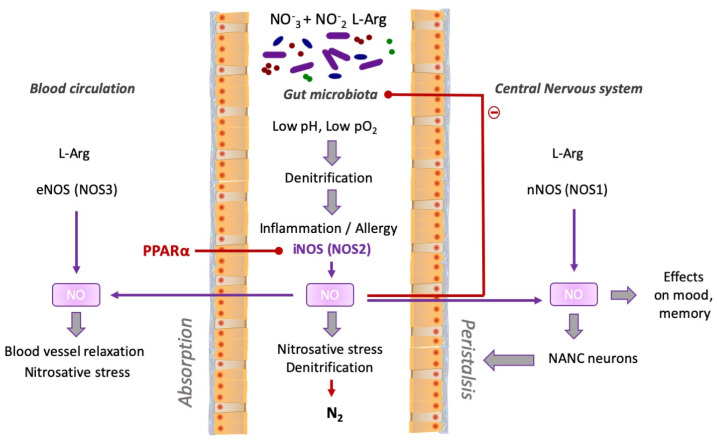
The main sources of nitric oxide and its metabolism in the gut.

Finally, in the human host, NO may be generated enzymatically from L-arginine by NO synthases (NOS), namely NOS-1 (neuronal), NOS-2 (inducible, related to inflammation), and NOS-3 (endothelial) [105]; all of them may be expressed in the gut. NOS-1 in the special type of neurons that belong to the parasympathetic branch of the autonomous neural system, the so called NANC-neurons (non-adrenergic, non-cholinergic, sometimes called the “nitroegric” neurons), very important for the proper motor function (peristalsis) of the intestines [106]. NOS-2 is synthetized during inflammation in macrophages, neutrophils, and in other cells engaged in the inflammatory response [107]. NOS-3 is expressed in the blood vessel wall, in the endothelial cells, including the digestive tract vascularization, while the NO generated here causes the relaxation of blood vessels, leading to the decreased blood pressure and simultaneously to increase the blood volume in the circulation [108,109]. Therefore, because NO is the same, irrespectively of the source, modifications of its generation may modulate some of its functions (summarized in Figure 1).

Notably, PPARα and its agonists have been found to participate in the regulation of the activity of NOS, which should also affect the gut microbiota. In particular, both natural and synthetic PPARα agonists inhibit the expression of NOS2 gene, while facilitating NOS 1 and NOS 3 production. The stationary level of NO may affect the microbiota, as being a substrate for denitrification, but also as a strong antibacterial factor. Here, it should be noted that NOS in the absence of L-Arg turn into the effective producers of superoxide O_2_^−^. Superoxide anion is also generated during the neutrophil and macrophage oxidative burst, leading to the appearance of more toxic nitrosative agents, e.g., peroxinitrite, ONOO^−^ [110].

However, microbiota in general are sensitive even to low level of NO, and potential bacterial hosts use NO and its metabolites to destroy prokaryotic microorganisms [104]. These metabolites are mainly produced in the presence of oxygen, so the lack of oxygen prevents generation of toxic NO metabolites. This is probably another reason for the anaerobic induction of the “nitrate respiration” in the denitrifying bacteria. Moreover, many bacterial species are equipped with various systems to remove NO and protect their environment against the nitrosative shock. Besides the aforementioned NO reduction, the important protective enzymatic armamentarium includes antioxidative flavohemoglobins, controlled by Fur system (depending on the level of NO), and alkaline reductase of H_2_O_2_ present in *Salmonella typhimurium* or *Mycobacterium tuberculosis* [111,112].

At present, it has become obvious how important this set of reactions is for gut microbiota metabolism but also for the metabolism of the whole host organism. It has been shown that NO generated by oral microbiome may affect the systemic blood pressure [113,114]. The acute inflammation or hypersensitivity reactions (allergies, autoimmune conditions) contribute to the systemic balance of NO synthesis. The auto-aggression may lead to some types of diabetes by NO-related destruction of the Langerhans islets in the pancreas [115]. Currently, NO is regarded as factor of general importance in food allergies [116]. The effect of NO on the central nervous system is also not to be ignored. The anti-depressant, mood-controlling effects of NO have been described, as well as its positive impact on immediate memory [117,118].

As PPARs, including PPAR, have turned out to play an important role in the synthesis and utilization of NO [reviewed in [1], their resultant effect on the NO activity in the gut towards gut microbiota, while important, are particularly difficult to estimate. This phenomenon is only just at the onset of its full understanding, which is a driving force to investigate the role of NO in the metabolism, in the pathology and in the evolution of nitric oxide and its complexes.

## 8. PPARα and Intestinal Permeability

The intestinal epithelial cells are tightly connected by certain areas of the cell membrane referred to as tight junctions (TJs), the dynamic structures, actin cytoskeleton-bound and built by about forty structural and regulatory proteins. One of the important functions of intestinal mucosa involves creation of a physical barrier to the outer space, not allowing the food antigens, food-borne pathogens and commensals with their products to enter into the deeper layers of the mucosa and to translocate further into bloodstream and other tissues. Gut permeability depends both on the composition of mucus covering apical part of epithelial cells, and on intracellular and paracellular transport. To be transported intracellularly, chemical compounds or small particles either have to enter cells by binding to special cell membrane-located transporters or have to be lipid-soluble to freely cross phospholipid bilayer. Paracellular transport of water-soluble substances does not require specific transporters and, in epithelia, is highly regulated by the condition of the TJ complex. Such intestinal epithelial permeability may be substantially increased in many pathological situations, such as infection or dysbiosis, leading to the condition called “leaky gut” syndrome [119]. It is obvious that the molecular keys able to temporarily open the TJ must exist, but only one of them, called zonulin, has been discovered and characterized so far. Zonulin is a multidomain protein, the haptoglobin 2 precursor similar to one of *Vibrio cholerae* toxins (zot). It is secreted by mammalian enterocytes into the lumen, e.g., in response to stimulation by gut pathogens and gluten, and has been shown to be a molecular switch triggering TJ disassembly and an increase in epithelial permeability [120]. Zonulin (as its bacterial equivalent) binds to protease-activated receptor 2 (PAR2) on the enterocyte membrane and transactivates epidermal growth factor receptor (EGFR) route leading to phosphorylation of TJ proteins and disconnection of ZO-1 from tight junctional complex [107].

It has also been demonstrated that TJ rearrangement takes place and intestinal permeability increases upon production of proinflammatory cytokines in the gut (such as TNFα or IL-1β). The effect is driven by the activation of the canonical NF-κB pathway, myosin light chain kinase (MLCK) activation and degradation of occludin mRNA by miR-200c-3p [121].

What is more, the excessive shedding of mature enterocytes on the villi tips and failure of new TJ to be established quickly enough to prevent focal epithelial gaps is also a possible cause for intestinal barrier collapse [122]. The abnormal cell shedding has been observed in many gut pathologies [123].

Epithelial cells of a healthy gut operate in the steep oxygen gradient both in small intestine and, especially, in the colon. Enterocytes outside crypts are subjected to “physiological hypoxia” as their environment is characterized by much lower oxygen tension than elsewhere in the body (as low as 10 mmHg O_2_ in the colon) [124]. Hypoxia-evoked stabilization of hypoxia-inducible factors (HIF-1α and HIF-2) in the intestinal epithelium is linked to the maintenance of TJ organization and effective barrier formation by regulation of some TJ proteins such as claudin [125]. Simultaneously, complete local intestinal hypoxia (ischemia) related to various traumatic events lasting for prolonged period of time (the range of hours) and followed by reperfusion (I/R injury) is known to increase gut permeability both in vivo and in vitro in HIF-dependent manner (e.g., Caco-2 monolayer Trans Epithelial Resistance, TEER, is decreased upon experimental I/R procedure) [126]. The question emerges whether the activation of PPARα modulates intestinal barrier function connected to these two events: inflammation (sterile or infectious) and hypoxia. This concept has been partially addressed above (Section 2 and Section 3) in regard to the interaction between microbiota and gut mucosa.

### 8.1. PPARα Knock-out Mice and Intestinal Permeability

Some clues on the subject of PPARα modulation of intestinal barrier function came from the knock-out mice studies. In the research carried out by Mazzon and Cuzzocrea, *Ppara* wt (+/+) and knock-out (−/−) mice were subjected to dinitrobenzene sulfuric acid-evoked experimental colitis [127] or emotional stress in the form of immobilization [128]. It turned out that although in unstressed knock-out mice the gut permeability remained similar to the WT mice, the situation changed upon applying stressors. Both groups of animals responded to stress with increased gut permeability as measured by lactulose/mannitol double sugars permeability test (DSPT), but in *Ppara* −/− mice the barrier collapse were much more pronounced. It seems that lack of *Ppara* gene resulted in the significant augmentation of the tight junction structural rearrangement and the permeability rise upon stress. It could be concluded that PPARα might be able somehow to rescue impaired intestinal barrier.

### 8.2. PPARα Action on Intestinal Barrier Function and Inflammation

As the increase in gut permeability accompanies various systemic and local inflammatory diseases, including those of gastrointestinal tract [129], the question emerges whether the activation of PPARα signaling, being generally anti-inflammatory, can also directly or indirectly regulate gut permeability accompanying gut inflammation. There are some experimental results which strongly support this concept. In Caco-2 cells, the TNF-α treatment or high glucose present in the culture medium (up to 8 g/L) induced epithelial barrier defects (measured by microscopically assessed barrier tortuosity) and this phenomenon was reversed by the addition of fenofibrate. The effect of fenofibrate was abolished by the presence of PPARα antagonist GW6741 [96]. Fenofibrate was also proved to support PPARα-targeted recovery of the intestinal epithelial barrier in an animal model of diabetes mellitus (in dogs) [130].

It was also shown that during DSS-induced colitis in mice the level of two main tight junction proteins, occludin and tight junction protein-1 (Tijp1) in the distal colon was significantly reduced and these proteins were restored by treatment with the natural PPARα ligand, OEA [131]. Matrine, a naturally occurring alkaloid, a bioactive component of herbs, was shown to enhance the intestinal barrier of mice in the same model of colitis as analyzed by intestinal histopathology and by immunoblotting analysis of intestinal proteins, showing the increase in TJ proteins in the intestine. Interestingly, matrine simultaneously inhibited the PPAR-α signaling pathway in the gut [132].

### 8.3. PPARα Action on Intestinal Regeneration and Its Possible Influence on Gut Permeability

Genome-wide analysis of PPARα activation in murine villus cells of small intestinal epithelium revealed the downregulation of MYC and the cluster of other genes connected with cell proliferation, cell cycle and apoptosis (e.g., EGFR and CASP3) under the influence of PPARα activation (by Wy-16434 and fenofibrate treatment). It is concluded that this regulation leads to an increase in proliferation and a decrease in apoptosis in the villi tips, as confirmed by morphological analysis of the villi, which turned out to be longer in jejunal sections taken from animals treated with PPARα activator [2]. Paneth cells have recently been shown to be important for gut homeostasis not only as producer of antimicrobial peptides, but also as regulators of intestinal epithelium regeneration [133]. It is well established that intestinal permeability tends to increase with age [134]. As already mentioned above, due to reduced PPARα signaling, Paneth cells of aging mice and humans, secrete excessive amounts of Wnt pathway inhibitor Notum, impairing the intestinal stem cells of the crypts and intestinal regeneration. In the model of intestinal organoids PPARα antagonist GW6471 can mimic the aging process and decrease proliferation [27]. The influence of PPARα on the increase in the intestinal permeability due to impaired regeneration and pathological cell shedding has not been directly studied by these authors or elsewhere, but it seems to be an interesting direction for future research.

### 8.4. PPARα Action on Intestinal Permeability and Hypoxia

The activation of HIF signalling pathway was shown to strongly downregulate *Ppara* expression in the colon epithelial cells [135], in the endothelial cells of retina [136] and in many types of cancer cells [137]. PPARα acts as an endogenous inhibitor of HIF-1. It was demonstrated using partially HIF-1α-deficient mice in intestinal ischemia/reperfusion (I/R) injury model that HIF-1 activation played a substantial role in the mechanism of intestinal injury, leading to the loss of barrier function. HIF-1α deficiency prevented the induction of ileal mucosal NOS-2 protein level after injury [138]. It is known that NOS-2 is a HIF target gene [139]. Endogenous NO is able to stabilize HIF1 during hypoxia [140]. Such reciprocal links indicate that, theoretically, the balance between HIF, PPAR and NO signalling could be important for gut homeostasis, especially in the context of physiological hypoxia and ischemia; the notion also confirmed by some elegant series of experiments. In the in vitro studies with rat intestinal epithelial cells IEC-6, and also in the studies in vivo on mice subjected to intestinal I/R injury, it was shown that miRNA (miR-23a-5p) targeting PPARα exacerbated damage by promoting oxidative stress. It was also evidenced that IEC cells can be rescued from injury by transfection with *Ppara* overexpression plasmid [141]. In experiments carried out on Caco-2 human cell line, it was proven that basolateral application of PEA and another endocannabinoid noladin ether (NE), endogenous receptor ligands, prevent the monolayer permeability response to oxygen deprivation through PPARα activation. However, the basolateral application of OEA acted in a reverse way, also via PPARα activation. Interestingly, palmitoylethanolamide (PEA) did not reduce pro-inflammatory cytokine secretion in Caco-2 cells, which suggested that the positive effects of PEA on permeability were not related to an anti-inflammatory effect [126]. It is worth noting that in mice liver and HepG2 hepatoma cells, OEA was able to inhibit endoplasmic reticulum stress-associated cell apoptosis and protect liver from I/R Injury [142], so this molecular mechanism might be precisely tuned and is dependent on the type of cells and spatial location of the signal.

## 9. Conclusions and Perspectives

**Figure 2:**
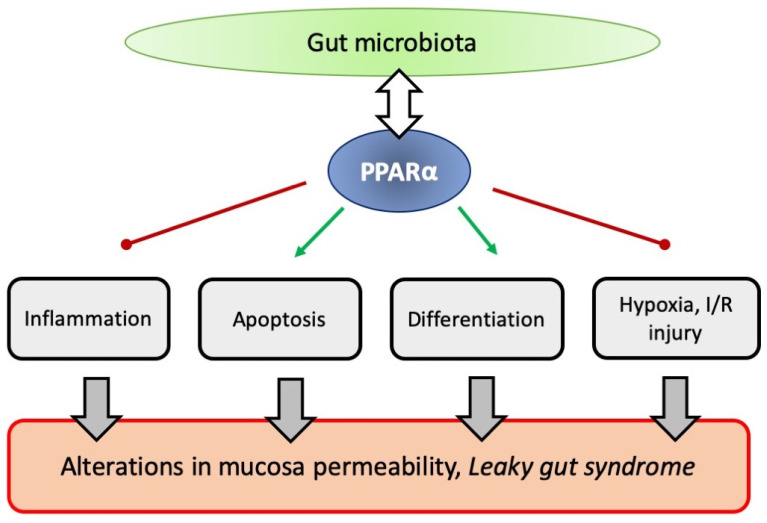
The scheme that summarizes the described impact of PPARα on various aspects of gut physiology, which may contribute to the development of the “leaky gut syndrome”.

PPARα is ubiquitously expressed through the mammalian gut, while its endogenous ligands (such as endocannabinoids) are secreted by intestines. However, the significance of this nuclear receptor in gastrointestinal homeostasis has only recently been undoubtedly spotted and it is still not fully understood, especially in the context of many pathologies which are believed to be gut-originated. Convincing evidence has recently been gathered indicating that PPARα activation plays a role in modulation of many important aspects of gut function, including microbiome interaction, cell regeneration, mucosal immunity, and intestinal permeability (as summarized in Figure 2). It seems that changes in PPARα signaling may contribute to the development of intestinal disorders, which is why PPARα activators are considered as potential new drugs (or rather old drugs in a new role), with respect to many diseases, particularly as a supplementary form of treatment.

The mammalian gastrointestinal tract is inhabited by plethora of commensal microorganisms, unique for the species, and changing from subject to subject and through personal life history. Here, we would like to stress the complication of the system and present the difficulties encountered by researchers conducting in vitro studies, the fact which makes the picture of microbiome-gut-PPARα axis still so non-obvious. Due to the profound influence of diverse microbial metabolites on PPARα and other closely intertwined signaling systems (e.g., NO signaling), it is very difficult to predict the therapeutic potential of PPARα activators in the gut. Nevertheless, it seems that, in near future, it will be a hot topic attracting a lot of attention in gut physiology. The possible interesting future directions for research include evaluation of PPARα agonists for treatment of the leaky gut symptoms, the gut mucosa regeneration and renewal (including functioning of a crypt stem cell niche), as well as analysis of the potential impact of clinically applied probiotic preparations on the PPARα signaling.

## Data Availability

Not applicable.

## Abbreviations

1-PG, 1-palmitoylglycerol; 2-PG, 2-palmitoylglycerol; ACOX, acyl-CoA oxidase; AP-1, activation protein 1; APCs, antigen presenting cells; ART, anti-retroviral therapy; BCR, B-cell receptor; CB, cannabinoid receptors; CD, Crohn disease; CPT1A, carnitine palmitoyltransferase 1A; DCs, dendritic cells; DNBS, dinitrobenzene sulfonic acid; DSPT, double sugar permeability test; DSS, dextran sulfate sodium; EPR/ESR, electron spin resonance/electron paramagnetic resonance; F/B, Firmicutes to Bacteroidetes ratio; GALT, gut-associated lymphoid tissue; GF, germ-free; GLP-1, glucagon-like protein 1; GSEA, gene set enrichment analysis; HFD, high fat diet; HIF, hypoxia inducible factor; IBD, inflammatory bowel disease; IEC, intestinal epithelial cells; IL, interleukin; IPA, ingenuity pathway analysis; I/R, ischemia/reperfusion; IRF, interferon-regulated factor; ISCs, intestinal stem cells; JAK, Janus-activated kinase; JNK, c-Jun N-terminal kinase; KO, knock-out; LAB, lactic acid bacteria; LPS, lipopolysaccharide; MHC, major histocompatibility complex; MHC2TA, MHC class II transactivator; MLCK, myosin light chain kinase; MMP-7, matrix metaloproteinase 7; mTORC1, mammalian/mechanistic target of rapamycin complex 1; NAFLD, nonalcoholic fatty liver disease; NE, noladin ether; NFκB, nuclear factor κB; NO, nitric oxide; NOS, nitric oxide synthase; OEA, oleylethanolamide; oxLDL, oxidized low density lipoprotein fraction; PEA, palmitoylethanolamide; PG, prostaglandin; PGC-1^α^, peroxisome proliferator-activated receptor γ coactivator 1α; PPAR, peroxisome proliferator-activated receptor α; PPI, protein-protein interactome; PPRE, peroxisome proliferator response element; RER, respiratory exchange ratio; ROR, retinoid orphan receptor; ROS, reactive oxygen species; RXR, retinoid X receptor; SCFA, short chain fatty acids; SFB, segmented filamentous bacteria; STAT, signal transducer and activator of transcription; Tijp1, tight junction protein 1; TJs, tight junctions; TLR, Toll-like receptors; TNF, tumor necrosis factor; UC, ulcerative colitis; ZO-1, *zonula occludens* protein 1.

## References

[1] Grabacka M., Pierzchalska M., Płonka P.M., Pierzchalski P. (2021). The Role of PPAR Alpha in the Modulation of Innate Immunity. Int. J. Mol. Sci..

[2] Bünger M., Bosch H.M.V.D., Van Der Meijde J., Kersten S., Hooiveld G.J.E.J., Muller M. (2007). Genome-wide analysis of PPARα activation in murine small intestine. Physiol. Genom..

[3] Luo Y., Xie C., Brocker C.N., Fan J., Wu X., Feng L., Wang Q., Zhao J., Lu D., Tandon M. (2019). Intestinal PPARα Protects Against Colon Carcinogenesis via Regulation of Methyltransferases DNMT1 and PRMT6. Gastroenterology.

[4] Hershberg R.M., Cho D.H., Youakim A., Bradley M.B., Lee J.S., Framson P.E., Nepom G. (1998). Highly polarized HLA class II antigen processing and presentation by human intestinal epithelial cells. J. Clin. Investig..

[5] Snoeck V., Goddeeris B., Cox E. (2005). The role of enterocytes in the intestinal barrier function and antigen uptake. Microbes Infect..

[6] Strobel S., Mowat A.M. (1998). Immune responses to dietary antigens: Oral tolerance. Immunol. Today.

[7] Mueller D., Jenkins M., Schwartz R.H. (1989). Clonal Expansion Versus Functional Clonal Inactivation: A Costimulatory Signalling Pathway Determines the Outcome of T Cell Antigen Receptor Occupancy. Annu. Rev. Immunol..

[8] Chen Y., Inobe J., Weiner H.L. (1995). Induction of oral tolerance to myelin basic protein in CD8-depleted mice: Both CD4+ and CD8+ cells mediate active suppression. J. Immunol..

[9] Lelouard H., Fallet M., de Bovis B., Méresse S., Gorvel J. (2012). Peyer’s Patch Dendritic Cells Sample Antigens by Extending Dendrites Through M Cell-Specific Transcellular Pores. Gastroenterology.

[10] Jakobsen M.A., Petersen R.K., Kristiansen K., De Lange M., Lillevang S.T. (2006). Peroxisome Proliferator-Activated Receptor alpha, delta, gamma1 and gamma2 Expressions are Present in Human Monocyte-Derived Dendritic Cells and Modulate Dendritic Cell Maturation by Addition of Subtype-Specific Ligands. Scand. J. Immunol..

[11] Shi H.-Y., Ge J.-B., Fang W.-Y., Yao K., Sun A.-J., Huang R.-C., Jia Q.-Z., Wang K.-Q., Zou Y.-Z., Cao X.-T. (2008). Peroxisome proliferator-activated receptor α agonist attenuates oxidized-low density lipoprotein induced immune maturation of human monocyte-derived dendritic cells. Chin. Med. J..

[12] Aleshin S., Reiser G. (2013). Role of the peroxisome proliferator-activated receptors (PPAR)-α, β/δ and γ triad in regulation of reactive oxygen species signaling in brain. Biol. Chem..

[13] Azuma Y.-T., Nishiyama K., Matsuo Y., Kuwamura M., Morioka A., Nakajima H., Takeuchi T. (2010). PPARα contributes to colonic protection in mice with DSS-induced colitis. Int. Immunopharmacol..

[14] Basso P.J., Sales-Campos H., Nardini V., Duarte-Silva M., Alves V.B.F., Bonfá G., Rodrigues C.C., Ghirotto B., Chica J.E.L., Nomizo A. (2021). Peroxisome Proliferator-Activated Receptor Alpha Mediates the Beneficial Effects of Atorvastatin in Experimental Colitis. Front. Immunol..

[15] Katkar G.D., Sayed I.M., Anandachar M.S., Castillo V., Vidales E., Toobian D., Usmani F., Sawires J.R., Leriche G., Yang J. (2022). Artificial intelligence-rationalized balanced PPARα/γ dual agonism resets dysregulated macrophage processes in inflammatory bowel disease. Commun. Biol..

[16] Lee J.W., Bajwa P.J., Carson M.J., Jeske D.R., Cong Y., Elson C.O., Lytle C., Straus D.S. (2007). Fenofibrate Represses Interleukin-17 and Interferon-γ Expression and Improves Colitis in Interleukin-10–Deficient Mice. Gastroenterology.

[17] Otagiri S., Ohnishi S., Ohara M., Fu Q., Yamamoto K., Yamamoto K., Katsurada T., Sakamoto N. (2020). Oleoylethanolamide Ameliorates Dextran Sulfate Sodium-Induced Colitis in Rats. Front. Pharmacol..

[18] Manoharan I., Suryawanshi A., Hong Y., Ranganathan P., Shanmugam A., Ahmad S., Swafford D., Manicassamy B., Ramesh G., Koni P. (2016). Homeostatic PPARα Signaling Limits Inflammatory Responses to Commensal Microbiota in the Intestine. J. Immunol..

[19] Riccardi L., Mazzon E., Bruscoli S., Esposito E., Crisafulli C., Di Paola R., Caminiti R., Riccardi C., Cuzzocrea S. (2009). Peroxisome proliferator-activated receptor-α modulates the anti-inflammatory effect of glucocorticoids in a model of inflammatory bowel disease in mice. Shock.

[20] Qi Y., Jiang C., Tanaka N., Krausz K.W., Brocker C.N., Fang Z.-Z., Bredell B.X., Shah Y.M., Gonzalez F.J. (2014). PPARα-dependent exacerbation of experimental colitis by the hypolipidemic drug fenofibrate. Am. J. Physiol. Liver Physiol..

[21] Zhou X., Cao L., Jiang C., Xie Y., Cheng X., Krausz K.W., Qi Y., Sun L., Shah Y.M., Gonzalez F.J. (2014). PPARα-UGT axis activation represses intestinal FXR-FGF15 feedback signalling and exacerbates experimental colitis. Nat. Commun..

[22] Gu X., Song Y., Chai Y., Lu F., Gonzalez F.J., Fan G., Qi Y. (2015). GC-MS metabolomics on PPARα-dependent exacerbation of colitis. Mol. BioSyst..

[23] Papamichael K., Lin S., Moore M., Papaioannou G., Sattler L., Cheifetz A.S. (2019). Infliximab in inflammatory bowel disease. Ther. Adv. Chronic Dis..

[24] Ann S.-J., Chung J.H., Park B.H., Kim S.H., Jang J., Park S., Kang S.-M., Lee S.-H. (2015). PPARα agonists inhibit inflammatory activation of macrophages through upregulation of β-defensin 1. Atherosclerosis.

[25] Peyrin-Biroulet L., Beisner J., Wang G., Nuding S., Oommen S.T., Kelly D., Parmentier-Decrucq E., Dessein R., Merour E., Chavatte P. (2010). Peroxisome proliferator-activated receptor gamma activation is required for maintenance of innate antimicrobial immunity in the colon. Proc. Natl. Acad. Sci. USA.

[26] Muniz L.R., Knosp C., Yeretssian G. (2012). Intestinal antimicrobial peptides during homeostasis, infection, and disease. Front. Immunol..

[27] Pentinmikko N., Iqbal S., Mana M., Andersson S., Cognetta A.B., Suciu R.M., Roper J., Luopajärvi K., Markelin E., Gopalakrishnan S. (2019). Notum produced by Paneth cells attenuates regeneration of aged intestinal epithelium. Nat. Cell Biol..

[28] Sengupta S., Peterson T.R., Laplante M., Oh S., Sabatini D.M. (2010). mTORC1 controls fasting-induced ketogenesis and its modulation by ageing. Nature.

[29] Varnat F., Heggeler B.B.-T., Grisel P., Boucard N., Corthésy–Theulaz I., Wahli W., Desvergne B. (2006). PPARβ/δ Regulates Paneth Cell Differentiation Via Controlling the Hedgehog Signaling Pathway. Gastroenterology.

[30] Zoetendal E.G., Raes J., van den Bogert B., Arumugam M., Booijink C.C.G.M., Troost F.J., Bork P., Wels M., De Vos W.M., Kleerebezem M. (2012). The human small intestinal microbiota is driven by rapid uptake and conversion of simple carbohydrates. ISME J..

[31] Zheng Y., Valdez P.A., Danilenko D.M., Hu Y., Sa S.M., Gong Q., Abbas A.R., Modrusan Z., Ghilardi N., De Sauvage F.J. (2008). Interleukin-22 mediates early host defense against attaching and effacing bacterial pathogens. Nat. Med..

[32] Tsai P.-Y., Zhang B., He W.-Q., Zha J.-M., Odenwald M.A., Singh G., Tamura A., Shen L., Sailer A., Yeruva S. (2017). IL-22 Upregulates Epithelial Claudin-2 to Drive Diarrhea and Enteric Pathogen Clearance. Cell Host Microbe.

[33] Salzman N.H., Hung K., Haribhai D., Chu H., Karlsson-Sjöberg J., Amir E., Teggatz P., Barman M., Hayward M., Eastwood D. (2010). Enteric defensins are essential regulators of intestinal microbial ecology. Nat. Immunol..

[34] Flannigan K.L., Denning T.L. (2018). Segmented filamentous bacteria-induced immune responses: A balancing act between host protection and autoimmunity. Immunology.

[35] Hedblom G.A., Reiland H.A., Sylte M.J., Johnson T.J., Baumler D.J. (2018). Segmented Filamentous Bacteria—Metabolism Meets Immunity. Front. Microbiol..

[36] Godlewski G., Offertáler L., Wagner J.A., Kunos G. (2009). Receptors for acylethanolamides—GPR55 and GPR119. Prostaglandins Other Lipid Mediat..

[37] Ryberg E., Larsson N., Sjögren S., Hjorth S., Hermansson N.-O., Leonova J., Elebring T., Nilsson K., Drmota T., Greasley P.J. (2007). The orphan receptor GPR55 is a novel cannabinoid receptor. J. Cereb. Blood Flow Metab..

[38] Lauffer L.M., Iakoubov R., Brubaker P.L. (2009). GPR119 Is Essential for Oleoylethanolamide-Induced Glucagon-Like Peptide-1 Secretion From the Intestinal Enteroendocrine L-Cell. Diabetes.

[39] Petersen G., Sørensen C., Schmid P.C., Artmann A., Tang-Christensen M., Hansen S.H., Larsen P.J., Schmid H.H., Hansen H.S. (2006). Intestinal levels of anandamide and oleoylethanolamide in food-deprived rats are regulated through their precursors. Biochim. Biophys. Acta (BBA) Mol. Cell Biol. Lipids.

[40] LoVerme J., La Rana G., Russo R., Calignano A., Piomelli D. (2005). The search for the palmitoylethanolamide receptor. Life Sci..

[41] Igarashi M., Iwasa K., Yoshikawa K. (2020). Feeding regulation by oleoylethanolamide synthesized from dietary oleic acid. Prostaglandins Leukot. Essent. Fat. Acids.

[42] Di Paola M., Bonechi E., Provensi G., Costa A., Clarke G., Ballerini C., De Filippo C., Passani M.B. (2018). Oleoylethanolamide treatment affects gut microbiota composition and the expression of intestinal cytokines in Peyer’s patches of mice. Sci. Rep..

[43] Ley R.E., Bäckhed F., Turnbaugh P., Lozupone C.A., Knight R.D., Gordon J.I. (2005). Obesity alters gut microbial ecology. Proc. Natl. Acad. Sci. USA.

[44] Ley R.E., Turnbaugh P.J., Klein S., Gordon J.I. (2006). Microbial Ecology: Human Gut Microbes Associated with Obesity. Nature.

[45] Turnbaugh P.J., Ley R.E., Mahowald M.A., Magrini V., Mardis E.R., Gordon J.I. (2006). An Obesity-Associated Gut Microbiome with Increased Capacity for Energy Harvest. Nature.

[46] Bäckhed F., Ding H., Wang T., Hooper L.V., Koh G.Y., Nagy A., Semenkovich C.F., Gordon J.I. (2004). The Gut Microbiota as an Environmental Factor That Regulates Fat Storage. Proc. Natl. Acad. Sci. USA.

[47] Wang X., Yu C., Liu X., Yang J., Feng Y., Wu Y., Xu Y., Zhu Y., Li W. (2022). Fenofibrate Ameliorated Systemic and Retinal Inflammation and Modulated Gut Microbiota in High-Fat Diet-Induced Mice. Front. Cell Infect. Microbiol..

[48] Miller T.L., Wolin M.J. (1996). Pathways of acetate, propionate, and butyrate formation by the human fecal microbial flora. Appl. Environ. Microbiol..

[49] Parada Venegas D., De La Fuente M.K., Landskron G., González M.J., Quera R., Dijkstra G., Harmsen H.J.M., Faber K.N., Hermoso M.A. (2019). Short Chain Fatty Acids (SCFAs)-Mediated Gut Epithelial and Immune Regulation and Its Relevance for Inflammatory Bowel Diseases. Front. Immunol..

[50] Lin T.-L., Shu C.-C., Chen Y.-M., Lu J.-J., Wu T.-S., Lai W.-F., Tzeng C.-M., Lai H.-C., Lu C.-C. (2020). Like Cures Like: Pharmacological Activity of Anti-Inflammatory Lipopolysaccharides From Gut Microbiome. Front. Pharmacol..

[51] Vasques-Monteiro I.M.L., Silva-Veiga F.M., Miranda C.S., Gonçalves C.B.D.A., Daleprane J.B., Souza-Mello V. (2021). A rise in Proteobacteria is an indicator of gut-liver axis-mediated nonalcoholic fatty liver disease in high-fructose-fed adult mice. Nutr. Res..

[52] Bona M.D., Torres C.H.d.M., Lima S.C.V.C., Morais A.H.D.A., Lima A.M., Maciel B.L.L. (2022). Intestinal Barrier Permeability in Obese Individuals with or without Metabolic Syndrome: A Systematic Review. Nutrients.

[53] Veiga F.M.S., Miranda C.S., Martins F.F., Daleprane J.B., Mandarim-De-Lacerda C.A., Souza-Mello V. (2020). Gut-liver axis modulation in fructose-fed mice: A role for PPAR-alpha and linagliptin. J. Endocrinol..

[54] Silva-Veiga F.M., Miranda C.S., Vasques-Monteiro I.M.L., Souza-Tavares H., Martins F.F., Daleprane J.B., Souza-Mello V. (2022). Peroxisome proliferator-activated receptor-alpha activation and dipeptidyl peptidase-4 inhibition target dysbiosis to treat fatty liver in obese mice. World J. Gastroenterol..

[55] Müller T., Finan B., Bloom S., D’Alessio D., Drucker D., Flatt P., Fritsche A., Gribble F., Grill H., Habener J. (2019). Glucagon-like peptide 1 (GLP-1). Mol. Metab..

[56] Kirjavainen P.V., Ouwehand A.C., Isolauri E., Salminen S.J. (1998). The ability of probiotic bacteria to bind to human intestinal mucus. FEMS Microbiol. Lett..

[57] Macfarlane S., Furrie E., Cummings J.H., Macfarlane G.T. (2004). Chemotaxonomic Analysis of Bacterial Populations Colonizing the Rectal Mucosa in Patients with Ulcerative Colitis. Clin. Infect. Dis..

[58] Di Cerbo A., Palmieri B., Aponte M., Morales-Medina J.C., Iannitti T. (2016). Mechanisms and therapeutic effectiveness of lactobacilli. J. Clin. Pathol..

[59] Pircalabioru G., Aviello G., Kubica M., Zhdanov A., Paclet M.-H., Brennan L., Hertzberger R., Papkovsky D., Bourke B., Knaus U.G. (2016). Defensive Mutualism Rescues NADPH Oxidase Inactivation in Gut Infection. Cell Host Microbe.

[60] Singh A.K., Hertzberger R.Y., Knaus U.G. (2018). Hydrogen peroxide production by lactobacilli promotes epithelial restitution during colitis. Redox Biol..

[61] Goto T., Kim Y.-I., Furuzono T., Takahashi N., Yamakuni K., Yang H.-E., Li Y., Ohue R., Nomura W., Sugawara T. (2015). 10-oxo-12(Z)-octadecenoic acid, a linoleic acid metabolite produced by gut lactic acid bacteria, potently activates PPARγ and stimulates adipogenesis. Biochem. Biophys. Res. Commun..

[62] Nagatake T., Kishino S., Urano E., Murakami H., Kitamura N., Konishi K., Ohno H., Tiwari P., Morimoto S., Node E. (2022). Intestinal microbe-dependent ω3 lipid metabolite αKetoA prevents inflammatory diseases in mice and cynomolgus macaques. Mucosal Immunol..

[63] Kishino S., Takeuchi M., Park S.-B., Hirata A., Kitamura N., Kunisawa J., Kiyono H., Iwamoto R., Isobe Y., Arita M. (2013). Polyunsaturated fatty acid saturation by gut lactic acid bacteria affecting host lipid composition. Proc. Natl. Acad. Sci. USA.

[64] Zheng J., Wittouck S., Salvetti E., Franz C.M.A.P., Harris H.M.B., Mattarelli P., O’Toole P.W., Pot B., Vandamme P., Walter J. (2020). A taxonomic note on the genus Lactobacillus: Description of 23 novel genera, emended description of the genus Lactobacillus Beijerinck 1901, and union of Lactobacillaceae and Leuconostocaceae. Int. J. Syst. Evol. Microbiol..

[65] Miyamoto J., Mizukure T., Park S.-B., Kishino S., Kimura I., Hirano K., Bergamo P., Rossi M., Suzuki T., Arita M. (2015). A Gut Microbial Metabolite of Linoleic Acid, 10-Hydroxy-cis-12-octadecenoic Acid, Ameliorates Intestinal Epithelial Barrier Impairment Partially via GPR40-MEK-ERK Pathway. J. Biol. Chem..

[66] Kim D.-H., Jeong D., Kang I.-B., Kim H., Song K.-Y., Seo K.-H. (2017). Dual function of *Lactobacillus kefiri* DH5 in preventing high-fat-diet-induced obesity: Direct reduction of cholesterol and upregulation of PPAR-α in adipose tissue. Mol. Nutr. Food Res..

[67] Nakamura F., Ishida Y., Aihara K., Sawada D., Ashida N., Sugawara T., Aoki Y., Takehara I., Takano K., Fujiwara S. (2016). Effect of fragmented *Lactobacillus amylovorus* CP1563 on lipid metabolism in overweight and mildly obese individuals: A randomized controlled trial. Microb. Ecol. Health Dis..

[68] Nakamura F., Ishida Y., Sawada D., Ashida N., Sugawara T., Sakai M., Goto T., Kawada T., Fujiwara S. (2016). Fragmented Lactic Acid Bacterial Cells Activate Peroxisome Proliferator-Activated Receptors and Ameliorate Dyslipidemia in Obese Mice. J. Agric. Food Chem..

[69] Cai H., Wen Z., Li X., Meng K., Yang P. (2020). Lactobacillus plantarum FRT10 alleviated high-fat diet–induced obesity in mice through regulating the PPARα signal pathway and gut microbiota. Appl. Microbiol. Biotechnol..

[70] Jiang J., Xiong J., Ni J., Chen C., Wang K. (2021). Live Combined B. subtilis and E. faecium Alleviate Liver Inflammation, Improve Intestinal Barrier Function, and Modulate Gut Microbiota in Mice with Non-Alcoholic Fatty Liver Disease. Med. Sci. Monit..

[71] Yang J.-Y., Lee Y.-S., Kim Y., Lee S.-H., Ryu S., Fukuda S., Hase K., Yang C.-S., Lim H.S., Kim M.-S. (2017). Gut commensal Bacteroides acidifaciens prevents obesity and improves insulin sensitivity in mice. Mucosal Immunol..

[72] Moosavi S.M., Sepahi A.A., Mousavi S.F., Vaziri F., Siadat S.D. (2020). The effect of Faecalibacterium prausnitzii and its extracellular vesicles on the permeability of intestinal epithelial cells and expression of PPARs and ANGPTL4 in the Caco-2 cell culture model. J. Diabetes Metab. Disord..

[73] Deleu S., Machiels K., Raes J., Verbeke K., Vermeire S. (2021). Short chain fatty acids and its producing organisms: An overlooked therapy for IBD?. eBioMedicine.

[74] Liu L., Li Q., Yang Y., Guo A. (2021). Biological Function of Short-Chain Fatty Acids and Its Regulation on Intestinal Health of Poultry. Front. Veter Sci..

[75] Higashimura Y., Naito Y., Takagi T., Uchiyama K., Mizushima K., Yoshikawa T. (2015). Propionate Promotes Fatty Acid Oxidation through the Up-Regulation of Peroxisome Proliferator-Activated Receptor α in Intestinal Epithelial Cells. J. Nutr. Sci. Vitaminol..

[76] Kasubuchi M., Hasegawa S., Hiramatsu T., Ichimura A., Kimura I. (2015). Dietary Gut Microbial Metabolites, Short-chain Fatty Acids, and Host Metabolic Regulation. Nutrients.

[77] Zhou L., Zhang M., Wang Y., Dorfman R.G., Liu H., Yu T., Chen X., Tang D., Xu L., Yin Y. (2018). Faecalibacterium prausnitzii Produces Butyrate to Maintain Th17/Treg Balance and to Ameliorate Colorectal Colitis by Inhibiting Histone Deacetylase 1. Inflamm. Bowel Dis..

[78] Sokol H., Seksik P., Furet J.P., Firmesse O., Nion-Larmurier I., Beaugerie L., Cosnes J., Corthier G., Marteau P., Doré J. (2009). Low counts of Faecalibacterium prausnitzii in colitis microbiota. Inflamm. Bowel Dis..

[79] Martinez-Medina M., Aldeguer X., Gonzalez-Huix F., Acero D., Garcia-Gil J.L. (2006). Abnormal microbiota composition in the ileocolonic mucosa of Crohnʼs disease patients as revealed by polymerase chain reaction-denaturing gradient gel electrophoresis. Inflamm. Bowel Dis..

[80] Frank D.N., St Amand A.L., Feldman R.A., Boedeker E.C., Harpaz N., Pace N.R. (2007). Molecular-phylogenetic characterization of microbial community imbalances in human inflammatory bowel diseases. Proc. Natl. Acad. Sci. USA.

[81] Dao M.C., Everard A., Aron-Wisnewsky J., Sokolovska N., Prifti E., Verger E.O., Kayser B.D., Levenez F., Chilloux J., Hoyles L. (2016). *Akkermansia muciniphila* and improved metabolic health during a dietary intervention in obesity: Relationship with gut microbiome richness and ecology. Gut.

[82] Everard A., Belzer C., Geurts L., Ouwerkerk J.P., Druart C., Bindels L.B., Guiot Y., Derrien M., Muccioli G.G., Delzenne N.M. (2013). Cross-talk between *Akkermansia muciniphila* and intestinal epithelium controls diet-induced obesity. Proc. Natl. Acad. Sci. USA.

[83] Collado M.C., Derrien M., Isolauri E., de Vos W.M., Salminen S. (2007). Intestinal Integrity and *Akkermansia muciniphila*, a Mucin-Degrading Member of the Intestinal Microbiota Present in Infants, Adults, and the Elderly. Appl. Environ. Microbiol..

[84] Derrien M., Van Baarlen P., Hooiveld G., Norin E., Müller M., de Vos W.M. (2011). Modulation of Mucosal Immune Response, Tolerance, and Proliferation in Mice Colonized by the Mucin-Degrader *Akkermansia muciniphila*. Front. Microbiol..

[85] Derrien M., Belzer C., de Vos W.M. (2017). *Akkermansia muciniphila* and its role in regulating host functions. Microb. Pathog..

[86] Reunanen J., Kainulainen V., Huuskonen L., Ottman N., Belzer C., Huhtinen H., de Vos W.M., Satokari R. (2015). *Akkermansia muciniphila* Adheres to Enterocytes and Strengthens the Integrity of the Epithelial Cell Layer. Appl. Environ. Microbiol..

[87] Shin N.R., Lee J.C., Lee H.Y., Kim M.S., Whon T.W., Lee M.S., Bae J.W. (2014). An increase in the *Akkermansia* spp. population induced by metformin treatment improves glucose homeostasis in diet-induced obese mice. Gut.

[88] Chelakkot C., Choi Y., Kim D.-K., Park H.T., Ghim J., Kwon Y., Jeon J., Kim M.-S., Jee Y.-K., Gho Y.S. (2018). *Akkermansia muciniphila*-derived extracellular vesicles influence gut permeability through the regulation of tight junctions. Exp. Mol. Med..

[89] Ashrafian F., Behrouzi A., Shahriary A., Badi S.A., Davari M., Khatami S., Jamnani F.R., Fateh A., Vaziri F., Siadat S.D. (2019). Comparative study of effect of *Akkermansia muciniphila* and its extracellular vesicles on toll-like receptors and tight junction. Gastroenterol. Hepatol. Bed Bench.

[90] Liu Y., Yang M., Tang L., Wang F., Huang S., Liu S., Lei Y., Wang S., Xie Z., Wang W. (2022). TLR4 regulates RORγt+ regulatory T-cell responses and susceptibility to colon inflammation through interaction with *Akkermansia muciniphila*. Microbiome.

[91] Earley H., Lennon G., Balfe A., Coffey J.C., Winter D.C., O’Connell P.R. (2019). The abundance of *Akkermansia muciniphila* and its relationship with sulphated colonic mucins in health and ulcerative colitis. Sci. Rep..

[92] Zhang T., Ji X., Lu G., Zhang F. (2021). The potential of *Akkermansia muciniphila* in inflammatory bowel disease. Appl. Microbiol. Biotechnol..

[93] Zhang T., Li P., Wu X., Lu G., Marcella C., Ji X., Ji G., Zhang F. (2020). Alterations of *Akkermansia muciniphila* in the inflammatory bowel disease patients with washed microbiota transplantation. Appl. Microbiol. Biotechnol..

[94] Depommier C., Vitale R.M., Iannotti F.A., Silvestri C., Flamand N., Druart C., Everard A., Pelicaen R., Maiter D., Thissen J.-P. (2021). Beneficial Effects of *Akkermansia muciniphila* Are Not Associated with Major Changes in the Circulating Endocannabinoidome but Linked to Higher Mono-Palmitoyl-Glycerol Levels as New PPARα Agonists. Cells.

[95] Plovier H., Everard A., Druart C., Depommier C., Van Hul M., Geurts L., Chilloux J., Ottman N., Duparc T., Lichtenstein L. (2017). A purified membrane protein from *Akkermansia muciniphila* or the pasteurized bacterium improves metabolism in obese and diabetic mice. Nat. Med..

[96] Crakes K.R., Rocha C.S., Grishina I., Hirao L.A., Napoli E., Gaulke C.A., Fenton A., Datta S., Arredondo J., Marco M.L. (2019). PPARα-targeted mitochondrial bioenergetics mediate repair of intestinal barriers at the host–microbe intersection during SIV infection. Proc. Natl. Acad. Sci. USA.

[97] Depommier C., Van Hul M., Everard A., Delzenne N.M., De Vos W.M., Cani P.D. (2020). Pasteurized *Akkermansia muciniphila* increases whole-body energy expenditure and fecal energy excretion in diet-induced obese mice. Gut Microbes.

[98] Payahoo L., Khajebishak Y., Alivand M.R., Soleimanzadeh H., Alipour S., Barzegari A., Ostadrahimi A. (2019). Investigation the effect of oleoylethanolamide supplementation on the abundance of *Akkermansia muciniphila* bacterium and the dietary intakes in people with obesity: A randomized clinical trial. Appetite.

[99] Crawford P.A., Crowley J.R., Sambandam N., Muegge B.D., Costello E.K., Hamady M., Knight R., Gordon J.I. (2009). Regulation of myocardial ketone body metabolism by the gut microbiota during nutrient deprivation. Proc. Natl. Acad. Sci. USA.

[100] Tiso M., Schechter A.N. (2015). Nitrate Reduction to Nitrite, Nitric Oxide and Ammonia by Gut Bacteria under Physiological Conditions. PLoS ONE.

[101] Pereira C., Ferreira N.R., Rocha B.S., Barbosa R.M., Laranjinha J. (2013). The redox interplay between nitrite and nitric oxide: From the gut to the brain. Redox Biol..

[102] Parham N.J., Gibson G.R. (2000). Microbes involved in dissimilatory nitrate reduction in the human large intestine. FEMS Microbiol. Ecol..

[103] Vermeiren J., Van de Wiele T., Verstraete W., Boeckx P., Boon N. (2009). Nitric Oxide Production by the Human Intestinal Microbiota by Dissimilatory Nitrate Reduction to Ammonium. J. Biomed. Biotechnol..

[104] Zumft W.G. (1997). Cell biology and molecular basis of denitrification. Microbiol. Mol. Biol. Rev..

[105] Alderton W., Cooper C., Knowles R.G. (2001). Nitric oxide synthases: Structure, function and inhibition. Biochem. J..

[106] Sanders K.M., Ward S.M. (2019). Nitric oxide and its role as a non-adrenergic, non-cholinergic inhibitory neurotransmitter in the gastrointestinal tract. Br. J. Pharmacol..

[107] Tripathi P., Tripathi P., Kashyap L., Singh V. (2007). The role of nitric oxide in inflammatory reactions. FEMS Immunol. Med. Microbiol..

[108] Fukumura D., Gohongi T., Kadambi A., Izumi Y., Ang J., Yun C.-O., Buerk D.G., Huang P.L., Jain R.K. (2001). Predominant role of endothelial nitric oxide synthase in vascular endothelial growth factor-induced angiogenesis and vascular permeability. Proc. Natl. Acad. Sci. USA.

[109] Chen K., Pittman R.N., Popel A. (2008). Nitric Oxide in the Vasculature: Where Does It Come From and Where Does It Go? A Quantitative Perspective. Antioxidants Redox Signal..

[110] Beckman J.S., Koppenol W.H. (1996). Nitric oxide, superoxide, and peroxynitrite: The good, the bad, and ugly. Am. J. Physiol. Cell Physiol..

[111] Forrester M.T., Foster M.W. (2012). Protection from nitrosative stress: A central role for microbial flavohemoglobin. Free Radic. Biol. Med..

[112] Sasaki Y., Oguchi H., Kobayashi T., Kusama S., Sugiura R., Moriya K., Hirata T., Yukioka Y., Takaya N., Yajima S. (2016). Nitrogen oxide cycle regulates nitric oxide levels and bacterial cell signaling. Sci. Rep..

[113] Cookson T.A. (2021). Bacterial-Induced Blood Pressure Reduction: Mechanisms for the Treatment of Hypertension via the Gut. Front. Cardiovasc. Med..

[114] Bryan N.S., Tribble G., Angelov N. (2017). Oral Microbiome and Nitric Oxide: The Missing Link in the Management of Blood Pressure. Curr. Hypertens. Rep..

[115] Spinas G.A. (1999). The Dual Role of Nitric Oxide in Islet β-Cells. Physiology.

[116] Raithel M., Hagel A.F., Zopf Y., Bijlsma P.B., De Rossi T.M., Gabriel S., Weidenhiller M., Kressel J., Hahn E.G., Konturek P.C. (2012). Analysis of immediate ex vivo release of nitric oxide from human colonic mucosa in gastrointestinally mediated allergy, inflammatory bowel disease and controls. J. Physiol. Pharmacol. Off. J. Pol. Physiol. Soc..

[117] Taheri P., Mohammadi F., Nazeri M., Zarei M.R., Chamani G., Esfahlani M.A., Taheri F., Shabani M. (2020). Nitric oxide role in anxiety-like behavior, memory and cognitive impairments in animal model of chronic migraine. Heliyon.

[118] Joca S.R.L., Sartim A.G., Roncalho A.L., Diniz C.F.A., Wegener G. (2019). Nitric oxide signalling and antidepressant action revisited. Cell Tissue Res..

[119] An J., Liu Y., Wang Y., Fan R., Hu X., Zhang F., Yang J., Chen J. (2022). The Role of Intestinal Mucosal Barrier in Autoimmune Disease: A Potential Target. Front. Immunol..

[120] Fasano A. (2020). All disease begins in the (leaky) gut: Role of zonulin-mediated gut permeability in the pathogenesis of some chronic inflammatory diseases. F1000Research.

[121] Kaminsky L.W., Al-Sadi R., Ma T.Y. (2021). IL-1β and the Intestinal Epithelial Tight Junction Barrier. Front. Immunol..

[122] Ngo P.A., Neurath M.F., López-Posadas R. (2022). Impact of Epithelial Cell Shedding on Intestinal Homeostasis. Int. J. Mol. Sci..

[123] Williams J.M., Duckworth C.A., Burkitt M.D., Watson A.J.M., Campbell B.J., Pritchard D.M. (2015). Epithelial cell shedding and barrier function: A matter of life and death at the small intestinal villus tip. Vet. Pathol..

[124] Glover L.E., Lee J.S., Colgan S.P. (2016). Oxygen metabolism and barrier regulation in the intestinal mucosa. J. Clin. Investig..

[125] Saeedi B.J., Kao D.J., Kitzenberg D.A., Dobrinskikh E., Schwisow K.D., Masterson J.C., Kendrick A.A., Kelly C.J., Bayless A.J., Kominsky D.J. (2015). HIF-dependent regulation of claudin-1 is central to intestinal epithelial tight junction integrity. Mol. Biol. Cell.

[126] Karwad M., Couch D., Wright K., Tufarelli C., Larvin M., Lund J., O’Sullivan S. (2019). Endocannabinoids and endocannabinoid-like compounds modulate hypoxia-induced permeability in CaCo-2 cells via CB1, TRPV1, and PPARα. Biochem. Pharmacol..

[127] Mazzon E., Cuzzocrea S. (2007). Absence of functional peroxisome proliferator-activated receptor-α enhanced ileum permeability during experimental colitis. Shock.

[128] Mazzon E., Cuzzocrea S. (2008). Role of TNF-α in ileum tight junction alteration in mouse model of restraint stress. Am. J. Physiol. Liver Physiol..

[129] Fukui H. (2016). Increased Intestinal Permeability and Decreased Barrier Function: Does It Really Influence the Risk of Inflammation?. Inflamm. Intest. Dis..

[130] Crakes K.R., Pires J., Quach N., Ellis-Reis R.E., Greathouse R., Chittum K.A., Steiner J.M., Pesavento P., Marks S.L., Dandekar S. (2021). Fenofibrate promotes PPARα-targeted recovery of the intestinal epithelial barrier at the host-microbe interface in dogs with diabetes mellitus. Sci. Rep..

[131] Lama A., Provensi G., Amoriello R., Pirozzi C., Rani B., Mollica M.P., Raso G.M., Ballerini C., Meli R., Passani M.B. (2020). The anti-inflammatory and immune-modulatory effects of OEA limit DSS-induced colitis in mice. Biomed. Pharmacother..

[132] Yao H., Shi Y., Yuan J., Sa R., Chen W., Wan X. (2021). Matrine protects against DSS-induced murine colitis by improving gut barrier integrity, inhibiting the PPAR-α signaling pathway, and modulating gut microbiota. Int. Immunopharmacol..

[133] Sato T., Van Es J.H., Snippert H.J., Stange D.E., Vries R.G., van den Born M., Barker N., Shroyer N.F., Van De Wetering M., Clevers H. (2011). Paneth cells constitute the niche for Lgr5 stem cells in intestinal crypts. Nature.

[134] Franceschi C., Campisi J. (2014). Chronic Inflammation (Inflammaging) and Its Potential Contribution to Age-Associated Diseases. J. Gerontol. A Ser. Biol. Sci. Med. Sci..

[135] Narravula S., Colgan S.P. (2001). Hypoxia-Inducible Factor 1-Mediated Inhibition of Peroxisome Proliferator-Activated Receptor α Expression During Hypoxia. J. Immunol..

[136] Wang Z., Moran E., Ding L., Cheng R., Xu X., Ma J.-X. (2014). PPARα Regulates Mobilization and Homing of Endothelial Progenitor Cells Through the HIF-1α/SDF-1 Pathway. Investig. Opthalmology Vis. Sci..

[137] Zhou J., Zhang S., Xue J., Avery J., Wu J., Lind S.E., Ding W.-Q. (2012). Activation of Peroxisome Proliferator-activated Receptor α (PPARα) Suppresses Hypoxia-inducible Factor-1α (HIF-1α) Signaling in Cancer Cells. J. Biol. Chem..

[138] Kannan K.B., Colorado I., Reino D., Palange D., Lu Q., Qin X., Abungu B., Watkins A., Caputo F.J., Xu D.-Z. (2011). Hypoxia-inducible factor plays a gut-injurious role in intestinal ischemia reperfusion injury. Am. J. Physiol. Liver Physiol..

[139] Matrone C., Pignataro G., Molinaro P., Irace C., Scorziello A., Di Renzo G.F., Annunziato L. (2004). HIF-1alpha reveals a binding activity to the promoter of iNOS gene after permanent middle cerebral artery occlusion. J. Neurochem..

[140] Chowdhury R., Godoy L.C., Thiantanawat A., Trudel L.J., Deen W.M., Wogan G.N. (2012). Nitric Oxide Produced Endogenously Is Responsible for Hypoxia-Induced HIF-1α Stabilization in Colon Carcinoma Cells. Chem. Res. Toxicol..

[141] Li L., Yin L.-H., Gao M., Xu L.-N., Qi Y., Peng J.-Y. (2020). MiR-23a-5p exacerbates intestinal ischemia–reperfusion injury by promoting oxidative stress via targeting PPAR alpha. Biochem. Pharmacol..

[142] Qi S., Yan Q., Wang Z., Liu D., Zhan M., Du J., Chen L. (2022). Oleoylethanolamide Alleviates Hepatic Ischemia-Reperfusion Injury via Inhibiting Endoplasmic Reticulum Stress-Associated Apoptosis. PPAR Res..

